# Improved Automated Radiosynthesis of [^11^C]PBR28

**DOI:** 10.3797/scipharm.1505-06

**Published:** 2015-06-19

**Authors:** Kiran Kumar Solingapuram Sai, Don Gage, Mike Nader, Robert H. Mach, Akiva Mintz

**Affiliations:** 1 Department of Radiology, Wake Forest School of Medicine, Medical Center Blvd, Winston-Salem, NC 27157, USA; 2 Department of Physiology and Pharmacology, Wake Forest School of Medicine, Medical Center Blvd, Winston-Salem, NC 27157, USA; 3 Department of Radiology, University of Pennsylvania, Philadelphia, PA 19104, USA

**Keywords:** Positron Emission Tomography (PET), Microglial activation, Synthesis, TSPO

## Abstract

Microglial activation is commonly identified by elevated levels of the 18 kDa translocator protein (TSPO) in response to several inflammatory processes. [^11^C]PBR28 is one of the most promising PET tracers to image TSPO in both human and non-human primates. In this study, we optimized the radiolabeling procedure of [^11^C]PBR28 for higher radiochemical yield, radiochemical purity, and specific activity, which can be easily translated to any automated module for clinical trials. Time-activity curves (TACs) derived from the dynamic PET imaging of male rhesus monkey brains demonstrated that [^11^C]PBR28 had suitable kinetics with radiotracer accumulation observed in the caudate, putamen, cerebellum, and frontal cortex region.

## Introduction

Expression of the peripheral benzodiazepine receptors (PBR), recently described as the 18 kDa translocator protein TSPO, is considered to be a hallmark for microglial activation [1, 2]. TSPOs are ubiquitous in active cerebral phagocytic cells and located in the outer mitochondrial membranes of peripheral organelles, including the brain, heart, kidney, liver, and lungs [3–5]. Several putative biological functions such as cell proliferation, cholesterol transportation, immune alterations, and apoptosis are associated with the transmembrane channels of the mitochondrial membrane, which are considered as a depository for TSPO [1, 6–10]. Significant levels of TSPO are observed during neuroinflammation, but absent in the resting microglial CNS parenchyma [2, 11–13]. Moreover, neurodegenerative disorders including Alzheimer’s disease (AD), Wernicke’s encephalopathy, epilepsy, Huntington’s disease, and cerebral ischemia have demonstrated increased TSPO in the cerebellum, olfactory lobes during disease development and progression stages, and have stimulated researchers to improve targeted neuroinflammation therapies [12–14]. However, accurate monitoring of the inflammatory processes related to each disease state will be required to determine if these therapies will be effective. Imaging microglial activation by targeting TSPO may provide an efficient index of the disease progression, and enhance the therapeutic planning for diseases affected by neuroinflammatory processes [15].

The TSPO targeting radiotracer, [^11^C]PK11195 has been used for the PET imaging of cerebral inflammation and was utilized to investigate microglial activation in a variety of animal models of neuroinflammation [16–21]. Additionally, [^11^C]PK11195 was evaluated in humans for neurological disease conditions such as multiple sclerosis, epilepsy, Parkinson’s disease, and AD for more than two decades [22-25]. However, despite its widespread use in neuroinflammation imaging, it demonstrates a low brain extraction, resulting in a low signal-to-noise ratio [18]. Additional studies have reported the accumulation of [^11^C]PK11195 in the regions of the brain that are not traditionally associated with disease processes resulting in low sensitivity, poor quantification, and the inability to image milder forms of neuroinflammation [26–30]. Moreover, several unidentified radiometabolites were observed after the administration of [^11^C]PK11195 [31, 32].

Recently, several new molecules have been developed to challenge the efficacy of [^11^C]PK11195 and to accurately quantify the TSPO density in humans. These include compounds containing benzothiazepines, indoleacetamides, alkaloids, benzoxapines, and aryloxyanilide derivatives which have been radiolabeled with [^11^C] and [^18^F] and evaluated as PET radiopharmaceuticals for neuroinflammation [33–35]. Additionally, Okuyamma *et al*. have reported several aryloxyanilide-based PET radioligands with high binding affinity and selectivity for TSPO. These radiotracers include [^11^C]PBR28, [^11^C]DAA1106, [^18^F]FMDAA1106, [^18^F]FEDAA1106, [^11^C]PBR01, and [^18^F]PBR06 and while some of these radiotracers demonstrated promising results, their viability in human subjects is either too sparse or unavailable to consider for clinical trials [36–41].

**Fig. 1:**
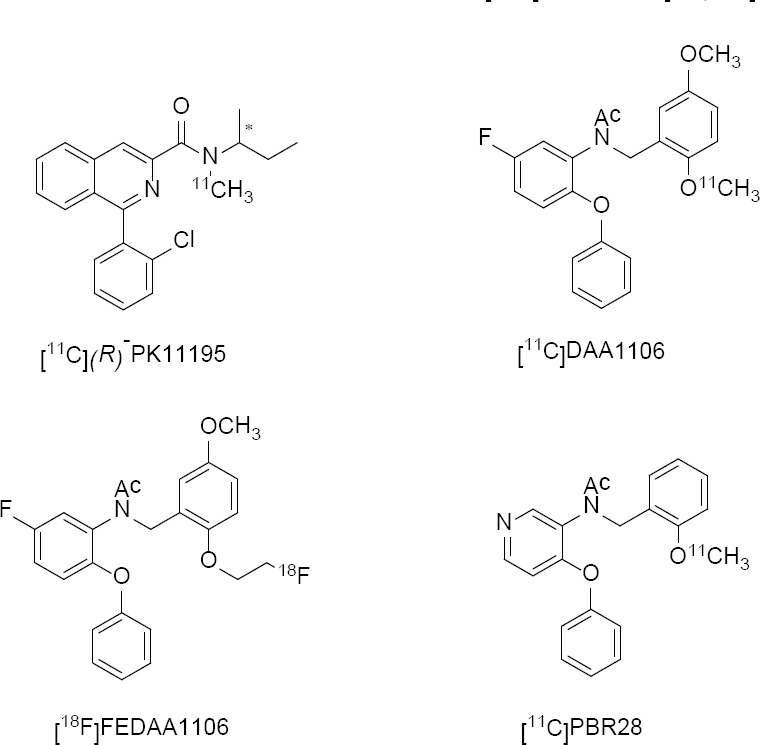
Chemical structures of some common aryloxyanilide-based TSPO radioligands

Figure 1 depicts several TSPO-targeting radioligands that have been thoroughly evaluated as neuroinflammation imaging radiopharmaceuticals. The aryloxyanilide-based tracers have received considerable attention and have been evaluated in both humans and non-human primates. *N*-(2-[^11^C]methoxybenzyl)-*N*-(4-phenoxypyridin-3-yl)acetamide, [^11^C]PBR28, is an aryloxyanilide-based TSPO targeting radiotracer synthesized by Imaizumi *et al*. [35, 40, 42, 43]. *In vitro*, it demonstrated a *K_i_* of 0.22 ± 0.03 nM and several blockade studies, including saturation autoradiography studies, homogenate and competition binding assays in human and monkey brains, were reported in greater detail using radioligand PK11195 to elucidate the binding specificity of [^11^C]PBR28 [37, 41, 44]. [^11^C]PBR28 also demonstrated excellent *in vivo* imaging properties [42, 43, 45]. Biodistribution studies and PET using [^11^C]PBR28 enabled researchers to accurately quantify cerebral artery inflammation caused by occlusion, and its pharmacokinetics in humans was concordant with the data obtained in non-human primate models [35, 46].

Recently, a lot of interest has been vested on investigating the imaging properties of [^11^C]PBR28 as a radiopharmaceutical to image TSPO in other organs and animal models [40–42]. Several new automation methods have been published recently for the synthesis [47–49]; however, the reported methods use a stronger methylation agent and base for the reaction, i.e. [^11^C]MeOTf as the methylating agent and sodium hydride as the base [47–49]. The radiochemistry method using [^11^C]MeOTf and sodium hydride (NaH, 60% in mineral oil) followed by sonication in acetonitrile does not seem to be a robust technique to get consistent radiochemical yields. Our study describes a simple route for the synthesis and radiochemical synthesis of [^11^C]PBR28 which resulted in a higher radiochemical yield over the previously reported methods [47–49] with high specific activity that can be easily adapted to any automated module, for example the most commonly used ones like GE FXC-pro, GE FXMeI-FXM, and TRASIS AIO modules. We used a simple reaction vial method to load the precursor in DMSO and then added a minimum of 1.9 µL of 5 N NaOH as the base. [^11^C]MeI was bubbled into the reaction mixture vial and was heated only to 60°C for 5 min. The radiochemical yield was ~45–55% and the specific activity ranged from 8000–9500 mCi/μmol (n=15, decay corrected to EOS).

### Radiochemical Synthesis of [^11^C]PBR28

[^11^C]MeI was produced in the Wake Forest PET Center Cyclotron facility on a GE PETtrace-800 Cyclotron. A nitrogen target containing 0.2% oxygen was irradiated for 15–20 min with a 50 µA beam of 16 MeV protons, to produce up to 1.5 Ci of [^11^C] CO_2_. The [^11^C]CO_2_ was converted to [^11^C]methane using a nickel catalyst [Shimalite-Ni (reduced), Japan P.N 221-27719] at 360°C using the GE PETrace MeI Microlab. [^11^C]methane was then reacted with gaseous iodine at 760°C to form [^11^C]MeI.

The radiochemical synthesis of [^11^C]PBR28 was carried out by alkylating the corresponding *p*-phenol precursor 6 with [^11^C]MeI in DMSO using NaOH as depicted in Scheme 2. Briefly, [^11^C]MeI was bubbled into the reaction vial containing precursor 6 (0.5 mg) in anhydrous DMSO (0.25 mL) and 5 N NaOH aqueous solution (1.9 μL) for 5 min. After the complete transfer of radioactivity, the sealed reaction vial was then heated at 60°C for 5 min. The reaction mixture was then quenched with HPLC mobile phase (1.0 mL) via the addition vial/loop. The radioactive reaction mixture was then injected onto a reversed-phase Phenomenex Prodigy C18 (250 × 10 mm, 10 μm) HPLC column to purify [^11^C]PBR28. The isocratic HPLC mobile phase solution consisted of 43% acetonitrile, 57% 0.1 M aqueous ammonium formate buffer solution (pH value 4.0–4.5) with a UV wavelength at 254 nm and a flow rate of 4.5 mL/min. The product [^11^C]PBR28 (R_t_ = 14.0-16.5 min) was collected into a vial containing Milli-Q water (50 mL), passed through a Sep-Pak C18 cartridge (Waters, Milford, MA) to trap the radiotracer [^11^C]PBR28, eluted from the cartridge with saline containing 10% absolute ethanol, and the eluting efficiency from the Sep-Pak C18 cartridge was ~80%. The final product was filtered using a sterile 0.22 µm pyrogen-free filter (Millipore Corp., Billerica, MA).

**Sch. 1:**
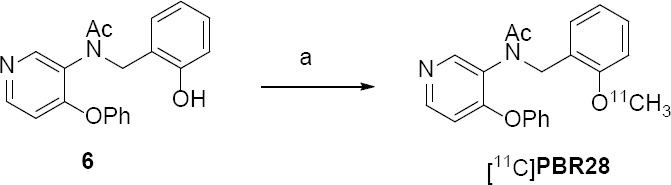
Reagents and Reaction conditions: a: [^11^C] CH_3_I, 5N NaOH, DMSO, 60°C, 5 min

**Sch. 1:**
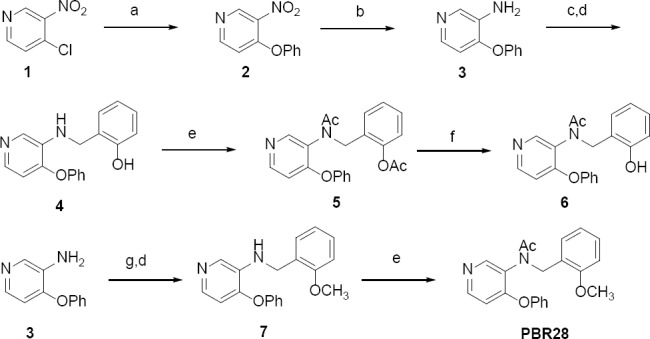
Reagents and Reaction conditions: a: PhOH, KOH/EtOH, reflux, 44%; b: SnCl_2_, EtOH, reflux, 99%; c: Salicylaldehyde, Toluene, reflux, 60%; d: NaBH_4_/MeOH, 85%; e: Acetylchloride, DCM, 85%; f: 1N LiOH/MeOH, 85%; g: 2-methoxybenzaldehyde, Toluene, reflux, 61%; d: NaBH_4_/MeOH, 60%.

### Quality Control Analysis of [^11^C]PBR28

[^11^C]PBR28 purity was assessed using an analytical reversed-phase Phenomenex Prodigy C18 analytical HPLC column (250 × 4.6 mm, 5 µm) and UV detection set at 254 nm. The mobile phase (1.5 mL/min) consisted of 70% acetonitrile and 30% 0.1 M aqueous ammonium formate pH 6.0–6.5 buffer solution. [^11^C]PBR28 showed retention at 6.0 min, and authentication of the product was performed with co-injection of the non-radioactive standard PBR28, which demonstrated similar retention times.

### Image Processing and Time-Activity Curves

PET PBR28 images were acquired on a General Electric 16-slice PET/CT Discovery ST Scanner which has 24 detector rings that provide 47 contiguous image planes over a maximum 70 cm transaxial field of view with CT attenuation correction. Axial spatial resolution of this scanner is 3.27 mm at the center of the gantry. Approximately 30 min prior to the scan, the monkey was anesthetized with ketamine (10 mg/kg, i.m.) and transported to the PET Center. Anesthesia was maintained during the scan by inhaled isoflurane (1.5%). The monkey was placed in the scanner and a catheter was inserted into an external vein for tracer injection and fluid replacement throughout the study. Body temperature was maintained at 40°C and vital signs (heart rate, blood pressure, respiration rate, and temperature) were monitored throughout the scanning procedure. An initial low dose CT-based attenuation correction scan was acquired. Next, [^11^C]PBR28 was injected and a 120-min dynamic acquisition scan was acquired. Thirty-three frames were acquired over 120 min (6 x 30 s, 3 x 60 s, 2 x 120 s, 22 x 300 s) in 3D mode (i.e., septa retracted). Image reconstruction of the 3D data was done using the 3D-reprojection method with full quantitative corrections. Emission data was corrected for attenuation and reconstructed into 128 × 128 matrices using a Hanning filter with a 4-mm cut-off transaxially and a ramp filter with an 8.5-mm cut-off axially. Data analysis was conducted using PMOD Biomedical Image Quantification Software (version 3.5; PMOD Technologies, Zurich, Switzerland). Brain uptake was defined by its standardized uptake value (SUV) calculated by dividing the tracer concentration in each pixel by the injected dose per body mass. ROIs for the basal ganglia and cerebellum were drawn and time-activity curves were generated.

## Results and Discussion

### Chemistry

Using a slight modification of the previously reported methods [48, 49], the [^11^C]PBR28 *p*-phenol precursor, **6**, and the corresponding nonradioactive standard, PBR28, were synthesized in higher chemical yields and a shorter reaction time (Scheme 2). Details of the synthesis have been provided in the “Supporting Information” folder.

Briefly, 4-chloro,3-nitro pyridine **1** underwent a base-assisted substitution reaction with phenol resulting in 3-nitro-4-phenoxypyridine, **2**, which upon reduction gave the corresponding amino compound **3**. The amine, **3**, was then subjected to a condensation reaction with salicylaldehyde, followed by an *in situ* NaBH_4_ reduction of the Schiff base to give the secondary amine, **4**. The amine, **4**, was then acetylated with acetyl chloride to give the diacetylated intermediate **5**, which upon selective LiOH-assisted *O*-deacetylation resulted in the corresponding [^11^C]PBR28 phenol precursor, **6**. The amine intermediate, **3**, was condensed with 2-methoxy-benzaldehyde to form the Schiff’s base *in situ*, which was reduced by NaBH_4_ to yield the secondary amine, compound **7**. Compound **7** was *N*-acetylated to give the non-radioactive standard, PBR28.

### Radiochemistry

The radiochemical synthesis of [^11^C]PBR28 was investigated using different reaction conditions (n=3) (Table 1). Alkylation reactions were carried out with both [^11^C]MeI and [^11^C]MeOTf; however, usage of the stronger alkylation agent [^11^C]MeOTf did not demonstrate any increase in the reaction yield and moreover, the production of [^11^C]MeI was comparatively easier and quicker over [^11^C]MeOTf. Several bases, including 5 N sodium hydroxide (NaOH) aqueous solution, 1 M cesium carbonate (Cs_2_CO_3_) aqueous solution, and tetra-*n*-butylammonium hydroxide (TBAH) aqueous solution (0.5 M & 1.0 M), were used as bases during the radiochemical synthesis. However, 5 N NaOH solution yielded [^11^C]PBR28 in higher radiochemical yields (RCY) and purity. Among the solvents dimethylsulfoxide (DMSO), dimethylformamide (DMF), and methylethylketone (MEK), DMSO was chosen as the suitable reaction solvent due to its high solubility property and high radioactivity trapping nature. The reaction temperature of 60°C was found to be suitable for the alkylation with [^11^C]MeI, as higher temperatures tend to form some undesirable radioactive side products, lowering the RCY. [^11^C]MeI, DMSO, 5 N NaOH, and 60°C were found to be the ideal methylating agent, solvent, base, and temperature, respectively, for the synthesis of [^11^C]PBR28.

**Tab. 1:**
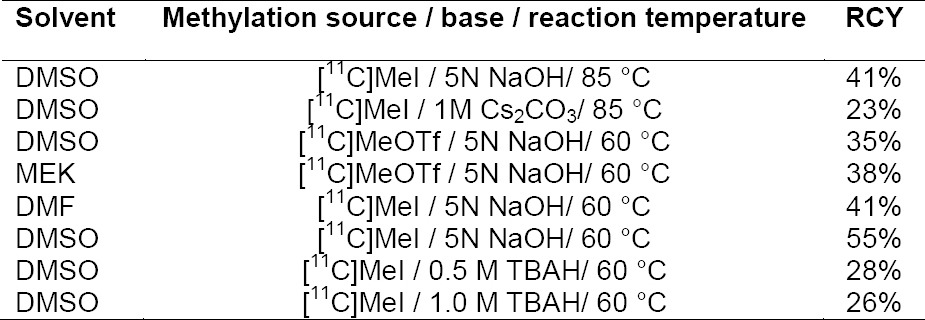
Reaction conditions and radiochemical yields for [^11^C]PBR28 (n=3) (decay corrected to End of Synthesis EOS)

Therefore, the radiolabeling of the [^11^C]PBR28 precursor was accomplished by the base-assisted *O*-alkylation technique for phenols, *i.e*. with the NaOH-MeI system. The radiolabeling reaction of the corresponding PBR28 precursor, 6, with [^11^C]MeI and aqueous 5.0 N NaOH in anhydrous DMSO at 60°C for 5.0 min, resulted in a higher radiochemical yield of approximately 45–55% (n=15) with the purity authenticated by co-injection of the non-radioactive standard, PBR28. Radiochemical synthesis, including the [^11^C]MeI transfer, reaction, HPLC purification, and radiotracer formulation for the monkey studies was completed within 28-32 min. The choice of the appropriate base was critical. In our hands, the use of 5 N NaOH led to the synthesis of [^11^C]PBR28 in a 45–55% radiochemical yield with >99% radiochemical purity, greater than has been previously reported in the literature [47, 48] and a specific activity of approximately 8000–9500 mCi/μmol (n=15, decay-corrected to EOS). Further, we optimized the radiolabeling procedure of [^11^C]PBR28 in GE-FXC, GE-FXMeI/FXM, and TRASIS AIO modules at the Wake Forest PET Center for clinical trials, using the least amount of 1.9 µL 5 N NaOH for the methylation step, to bring down the UV mass to <4.0 µg/mL in the final dose. Due to the improvement in the radiochemical yield, the synthesis of [^11^C]PBR28 can be easily translated to any automated radiochemistry modules around the world.

### Monkey PET Image Analysis

**Fig. 2:**
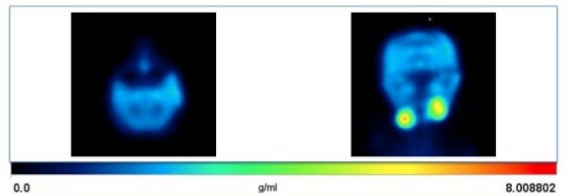
Representative PET images (left axial and right coronal) of [^11^C]PBR28 in a male monkey. The images were summed from 0–100 min after iv injection of 14 mCi [^11^C]PBR28

Dynamic small animal PET imaging was performed on male rhesus monkeys (n=3), which received an intravenous injection of [^11^C]PBR28. [^11^C]PBR28 demonstrated high radioactive uptakes in monkey brain regions, with greater activity localizing in the grey matter. Figure 2 represents the dynamic PET image of [^11^C]PBR28 (100 min) in a monkey brain.

Based on the TACs, the radioactivity accumulation reached a maximum approximately 10.0 min after the administration of [^11^C]PBR28, (n=3) (Figure 2). After 10 min post-injection of [^11^C]PBR28, the radioactive uptake obtained in the cerebellum was 0.1 (%ID/cc) and the basal ganglion was 0.091 (%ID/cc). Additionally, the [^11^C]PBR28 radioactive uptake pattern in these regions of the brain was consistent with cerebral TSPO distributions [35, 40, 44, 45]. Regional cerebral distribution of [^11^C]PBR28 was rather homogenous by the first 12 min after injection and no blatant accumulation was observed. As depicted by the TACs for the [^11^C]PBR28 tracer, initial uptake in the brain was satisfactory and by approximately 2 h p.i., the radioactivity was washed out from all regions of the brain. The lipophilicity of a tracer affects its binding and distribution and especially with brain-related tracer development, it is an important determinant of brain penetration (through the blood-brain barrier) [49, 50]. Clinical PET imaging studies, especially with C-11 PET probes, depend on tissue retention and clearance times [51]. From the TACs of [^11^C]PBR28, the pharmacokinetics are acceptable for probing the TSPO receptor density in the brain.

## Conclusion

In summary, we report the modified radiolabeling procedure for [^11^C]PBR28 with a high radiochemical yield, radiochemical purity, and specific activity to be directly translated and easily adapted to any automated modules for human injections and clinical trials. We further validated the radioactive uptakes of [^11^C]PBR28 in brains of male rhesus macaques using PET imaging studies, and [^11^C]PBR28 demonstrated favorable pharmacokinetics to image TSPO, which thus possesses a high potential to be a valuable *in vivo* PET tracer for imaging several neuroinflammatory processes, both in research and clinical settings. This study strongly reinforces the utility of the TSPO radiotracers to be further evaluated as neuroinflammation imaging agents.

## Experimental

### Chemistry

All reagents were purchased from Sigma-Aldrich and were used without additional purification. All reactions were carried out using anhydrous solvents unless otherwise stated. ^1^H-NMR was measured by the Varian^®^ 300 MHz NMR spectrometer and all chemical shifts are reported as ppm (δ). Melting points were measured using the Electrothermal Mel-Temp^®^ 3.0 melting point apparatus. All reactions were monitored by analytical thin-layer chromatography (TLC), and all UV-active spots were detected using the Mineralight® Lamp UVGL-25 UV lamp.

### PBR28 Synthesis

Based on the reported literature chemical methods [48, 49], the syntheses of radiolabeling precursor **6** and its non-radioactive standard PBR28 were carried out as depicted in Scheme 1 with slight modifications for better chemical yields and shorter reaction times.

#### 3-Nitro-4-phenoxypyridine (2)

KOH (1.27 g, 22.7 mmol) was added to a solution of phenol (2.13 g, 22.7 mmol) in anhydrous ethanol (50 mL) and refluxed for 30 min. 4-chloro-3-nitropyridine **1** (3.0 g, 18.9 mmol) was then added to this resulting mixture and refluxed for an additional 8 h. The reaction mixture was cooled to RT, evaporated under reduced pressure, washed with saturated NaHCO_3_ solution (30 mL), and extracted with ethylacetate (3 × 40 mL). The organic layers were combined, washed with brine solution (2 × 50 mL), and then dried over anhydrous Na_2_SO_4_. The solvent was filtered and concentrated under reduced pressure. Product **2** was isolated using silica gel column chromatography with a mixture of hexane: ethylacetate (1:1) as a white crystalline solid (1.8 g, 8.33 mmol, 44%), MP 72–76°C. ^1^H-NMR, CDCl_3_, δ: 9.04 (s, 1H), 8.46 (d, 1H, *J*=6 Hz), 7.43–7.38 (m, 2H), 7.29–7.24 (m, 1H), 7.09–7.06 (m, 2H), 6.69 (d, 1H, *J*=6 Hz).

#### 4-Phenoxypyridin-3-amine (3)

SnCl_2_.2H_2_O (9.2 g, 41 mmol) was added to a solution of **2** (1.7 g, 7.86 mmol) in ethanol (20 mL) and refluxed for 3 h. The reaction mixture was cooled to RT, quenched using ice-cold water (30 mL), basified with 50% NaOH to a pH~13, and then extracted with ethylacetate (3 × 60 mL). The organic layers were combined, washed, with saturated Na_2_CO_3_ solution (2 × 125 mL), followed with brine (2 × 200 mL), and then dried over anhydrous Na_2_SO_4_. The solvent was evaporated to give product **3** as a yellow solid (1.45 g, 7.78 mmol, 99%), MP 81–84°C. ^1^H-NMR, CDCl_3_, δ: 8.06 (s, 1H), 7.81 (d, 1H, *J* = 5.5 Hz), 7.35–7.29 (m, 2H), 7.16–7.10 (m, 1H), 7.01–6.98 (m, 2H), 6.49 (d, *J* = 5.4 Hz). 3.83 (b, 2H).

#### 2-{[(4-Phenoxypyridin-3-yl)amino]methyl}phenol (4)

Salicylaldehyde (0.62 mL, 5.90 mmol) was added to a solution of **3** (1.0 g, 5.37 mmol) in anhydrous toluene (20 mL) and refluxed using a Dean-Stark apparatus for 24 h. Excess toluene was evaporated under reduced pressure and the residue was then dissolved in methanol (50 mL). NaBH_4_ (0.81 g, 21.48 mmol) was added slowly to the above residue in methanol at 0°C, followed by stirring at RT for 1 h. The reaction mixture was further quenched with 5% aqueous acetic acid (17 mL) and extracted with ethylacetate (3 × 50 mL). The organic layers were combined and washed with saturated NaHCO_3_ solution (2 × 130 mL), brine (2 × 150 mL), and then evaporated to yield product **4** as a white crystalline solid (0.95 g, 3.24 mmol, 60%), MP 172–176°C and was used in the next step without additional purification. ^1^H-NMR, DMSO, δ: 9.53 (s, 1H), 7.62 (s, 1H), 7.61 (d, 1H, *J* = 5.1 Hz), 7.36 (t, 2H, *J* = 7.2 Hz), 7.16–6.94 (m, 5H), 6.73 (d, 1H, *J* = 7.5 Hz), 6.64 (t, 1H, *J* = 8.1 Hz), 6.45 (d, 1H, *J* = 5.4 Hz), 5.84 (t, 1H, *J* = 6.3 Hz), 4.25 (d, 2H, *J* = 6 Hz).

#### 2-{[Acetyl(4-phenoxypyridin-3-yl)amino]methyl}phenyl acetate (5)

Acetyl chloride (1.0 mL, 13.6 mmol) was slowly added to the solution of **4** (0.5 g, 1.711 mmol) in anhydrous DCM (15 mL) and was stirred at RT for 12 h. The reaction mixture was then quenched with saturated NaHCO_3_ solution (20 mL) and extracted with DCM (2 × 25 mL). The organic layers were combined, washed with brine (2 × 30 mL), and dried over anhydrous Na_2_SO_4_. The solvent was evaporated under reduced pressure and the crude material was purified using silica gel chromatography with ethylacetate to yield product **5** as a white crystalline solid (0.55 g, 1.46 mmol, 85%), MP 121–125°C. ^1^H-NMR, CDCl_3_, δ: 8.10 (d, 1H, *J* = 5.7 Hz), 7.18 (t, 2H, *J* = 6.5 Hz), 7.15–7.00 (m, 3H), (6.82 (q, 2H, *J* = 6.6 Hz), 6.38 (d, 1H, *J* = 5.7 Hz), 4.19 (d, 1H, *J* = 14.1 Hz), 4.59 (d, 1H, *J* = 14 Hz), 1.98 (s, 3H), 1.80 (s, 3H).

#### N-(2-Hydroxybenzyl)-N-(4-phenoxypyridin-3-yl)acetamide (6)

One N LiOH solution in methanol (15 mL) was added to a solution of **5** (0.5 g, 1.32 mmol) in anhydrous methanol (5.0 mL) and stirred at RT for 30 min. The reaction mixture was concentrated, quenched with water (20 mL), and extracted with ethylacetate (3 × 20 mL). The organic layers were combined and washed with brine solution (2 × 25 mL) and dried over anhydrous Na_2_SO_4_. The solvent was evaporated under reduced pressure to give product **6** as a white solid (0.37 g, 85%), MP 132–137°C. ^1^H-NMR, CDCl_3_, δ: 9.22 (s, 1H), 8.29 (d, 1H, *J* = 5.7 Hz), 8.20 (s, 1H), 7.31–7.26 (m, 2H), 7.18–7.07 (m, 2H), 6.81 (d, 1H, *J* = 8.4 Hz), 6.70–6.67 (m, 2H), 6.61–6.59 (m, 2H), 6.52 (d, 1H, *J* = 5.7 Hz), 4.71 (s, 2H), 1.91 (s, 3H).

#### N-(2-Methoxybenzyl)-4-phenoxypyridin-3-amine (7)

2-Methoxybenzaldehyde (0.80 mL, 5.90 mmol) was added to a solution of **3** (1.0 g, 5.37 mmol) in anhydrous toluene (20 mL) and refluxed using a Dean-Stark apparatus for 24 h. The reaction mixture was evaporated under reduced pressure and the residue was then dissolved in methanol (50 mL). NaBH_4_ (0.75 g, 19.8 mmol) was added slowly to the above residue in methanol at 0°C, followed by stirring at RT for 1 h. The reaction mixture was further quenched with 5% aqueous acetic acid (17 mL) and then extracted with ethylacetate (3 × 50 mL). The organic layers were combined, washed with saturated NaHCO_3_ solution (2 × 150 mL), brine (2 × 150 mL), and then concentrated to yield product **7** as a yellow thick oil (1.0 g, 3.26 mmol, 61%) and was used in the next step without any additional purification. ^1^H-NMR, CDCl_3_, δ: 8.02 (s, 1H), 7.76 (d, 1H, *J* = 5.7 Hz), 7.34–7.10 (m, 5H), 6.99 (d, 1H, *J* = 6.5 Hz), 6.96 (d, 1H, *J* = 6.5 Hz), 6.84 (q, 2H, *J* = 7.2 Hz), 6.46 (d, 1H, *J* = 5.5 Hz), 4.64 (br, 1H), 4.38 (s, 2H), 3.76 (s, 3H).

#### N-(2-Methoxybenzyl)-N-(4-phenoxypyridin-3-yl)acetamide (PBR28)

Acetyl chloride (2.0 mL, 26.11 mmol) was added slowly to a solution of **7** (1.0 g, 3.26 mmol) in anhydrous dichloromethane (15 mL) and stirred at RT for 24 h. The reaction mixture was quenched with saturated NaHCO_3_ solution (20 mL) and extracted with DCM (2 × 25 mL). The organic layers were combined and washed with brine solution (2 × 30 mL) and dried over anhydrous Na_2_SO_4_. The solvent was evaporated under reduced pressure and the product PBR28 was isolated after silica gel chromatography with ethylacetate as a white crystalline solid (1.02 g, 2.94 mmol, 90%), MP 89–93°C. ^1^H-NMR, CDCl_3_, δ: 8.15 (d, 1H, *J* = 5.7 Hz), 8.10 (s, 1H), 7.33–7.26 (m, 3H), 7.18–7.08 (m, 2H), 6.81–6.76 (m, 3H), 6.63 (d, 1H, *J* = 8.1 Hz), 6.45 (d, 1H, *J* = 5.7 Hz), 5.04 (d, 1H, *J* = 13.8 Hz), 4.79 (d, 1H, *J* = 14.1 Hz), 3.46 (s, 3H).

## Authors’ Statement

### Competing Interests

The authors declare no conflict of interest.
